# Identification of hub biomarkers and immune cell infiltration in polymyositis and dermatomyositis

**DOI:** 10.18632/aging.204098

**Published:** 2022-05-24

**Authors:** Si Chen, Haolong Li, Haoting Zhan, Xiaoli Zeng, Hui Yuan, Yongzhe Li

**Affiliations:** 1 Department of Clinical Laboratory, State Key Laboratory of Complex Severe and Rare Diseases, Peking Union Medical College Hospital, Chinese Academy of Medical Science and Peking Union Medical College, Beijing, China; 2 Department of Clinical Laboratory, Beijing Anzhen Hospital, Capital Medical University, Beijing, China

**Keywords:** idiopathic inflammatory myopathy, macrophage infiltration, interferon signaling, autoimmune disease, gene set expression analysis

## Abstract

Objective: Polymyositis (PM) and dermatomyositis (DM) are heterogeneous disorders. However, the etiology of PM/DM development has not been thoroughly clarified.

Methods: Gene expression data of PM/DM were obtained from Gene Expression Omnibus. We used robust rank aggregation (RRA) to identify differentially expressed genes (DEGs). Gene Ontology functional enrichment and pathway analyses were used to investigate potential functions of the DEGs. Weighted gene co-expression network analysis (WGCNA) was used to establish a gene co-expression network. CIBERSORT was utilized to analyze the pattern of immune cell infiltration in PM/DM. Protein–protein interaction (PPI) network, Venn, and association analyses between core genes and muscle injury were performed to identify hub genes. Receiver operating characteristic analyses were executed to investigate the value of hub genes in the diagnosis of PM/DM, and the results were verified using the microarray dataset GSE48280.

Results: Five datasets were included. The RRA integrated analysis identified 82 significant DEGs. Functional enrichment analysis revealed that immune function and the interferon signaling pathway were enriched in PM/DM. WGCNA outcomes identified MEblue and MEturquoise as key target modules in PM/DM. Immune cell infiltration analysis revealed greater macrophage infiltration and lower regulatory T-cell infiltration in PM/DM patients than in healthy controls. PPI network, Venn, and association analyses of muscle injury identified five putative hub genes: *TRIM22*, *IFI6*, *IFITM1*, *IFI35*, and *IRF9*.

Conclusions: Our bioinformatics analysis identified new genetic biomarkers of the pathogenesis of PM/DM. We demonstrated that immune cell infiltration plays a pivotal part in the occurrence of PM/DM.

## INTRODUCTION

The idiopathic inflammatory myopathies (IIMs) are characterized by muscle injury, with an annual incidence of 7.98 per million people, according to data from 1966 to 2013 [1]. IIMs include polymyositis (PM), dermatomyositis (DM), inclusion body myositis (IBD), juvenile dermatomyositis (JDM), anti-synthetase syndrome (ASS), and immune-mediated necrotizing myopathies (IMNM); historically, the two most common IIMs are PM and DM [2]. The disease progression and muscle impairments of PM and DM are similar, irrespective of the presence of skin lesions [3]. In addition, clinical studies have shown similar serological characteristics between PM and DM, especially with certain myositis-specific autoantibodies [4, 5]. Despite several decades of research, the exact pathogenesis of PM and DM remains unknown; their etiology may be a combination of multiple factors, such as immune activation, genetic background, and environmental factors [6].

In general, elucidating specific molecular pathways associated with a given disease would be of great significance in identifying disease subgroups, monitoring disease activity, and selecting treatment approaches. Studies of PM/DM-associated genes in the immune system and inflammatory response signaling pathways have also been reported, such as interferon-alpha (IFN-α) [7, 8], nuclear factor-κB (NF-κB) [9], IFN-γ [7, 8], tumor necrosis factor α (TNF-α) [10], toll-like receptors (TLRs) [11], and retinoic acid-inducible gene 1 (RIG-1) [12]. According to the literature, gene microarray technology has been extensively utilized to analyze gene expression in muscle or skin tissues of PM/DM patients. However, there are some contradictions regarding these microarray data [13–17] in reflecting the importance of IFN1-induced genes in the pathogenesis of DM and PM. These contradictions may be attributed to factors such as diverse analytic methods and platforms, inconsistent specimen quantity, and different sample sources.

Bioinformatics analysis is an efficient means of deep detection and mining of transcriptomic data. Furthermore, the robust rank aggregation (RRA) method has been utilized to discriminate variation in mRNA profiles between several datasets in diverse disease types, such as in tumors [18–20]. In this study, we included five mRNA microarray datasets from Gene Expression Omnibus (GEO) and used the RRA method to select differentially expressed genes (DEGs) between PM/DM and healthy controls. We next analyzed the molecular mechanisms of PM/DM using Gene Ontology (GO) functional enrichment analysis and Kyoto Encyclopedia of Genes and Genomes (KEGG) pathway analysis. In addition, other enrichment analyses such as gene set enrichment analysis (GSEA) and gene set variation analysis (GSVA) were used to study the molecular pathways of PM/DM. Weighted gene co-expression network analysis (WGCNA) was utilized to create a gene co-expression network and identify the most relevant modules in PM/DM. CIBERSORT analysis was utilized to estimate the various immune infiltrates in PM/DM. Protein–protein interaction (PPI) network analysis was performed to screen key genes, and Venn diagram analysis was used to assemble the DEGs of seven datasets. Subsequently, analysis of the correlation between core genes and muscle injury was conducted to indicate the potential functions of core genes in PM/DM. Finally, verification testing was carried out to identify five novel hub genes in the pathogenesis of PM/DM. We expect that the newly described DEGs and altered pathways between PM/DM and healthy controls found in this study may help to elucidate possible molecular mechanisms for their pathogenesis.

## MATERIALS AND METHODS

### Study design and data collection

Gene expression datasets were filtered using the GEO (http://www.ncbi.nlm.nih.gov/geo) database [21]. We comprehensively retrieved microarray studies using the keywords “Polymyositis,” “Dermatomyositis,” “Myositis,” “Gene expression,” “*Homo sapiens*,” and “Microarray.” Datasets were filtered using the next enrollment criteria: (1) including more than 10 specimens; (2) original information or gene expression analysis data obtainable in GEO. In light of the above criteria, GSE1551 [13], GSE3112 [22], GSE39454 [23], GSE46239, and GSE128470 [24] were finally recruited. Subsequently, the normalization and quality control of these data were executed with the “affy” R package [25]. We used the "sva" package [26] to eliminate the batch effect. The probes were transformed into homologous gene symbols using the R package “Rsubread” [27]. If several probes matched an identical sign, their average value was obtained. Further, genes without a corresponding genetic symbol were deleted.

### DEG screening

We established seven different groups for five GEO series: the GSE39454 series, which was split into GSE39454PM and GSE39454DM series; GSE128470, which was divided into GSE128470 PM and GSE128470 DM series. Data were analyzed using the “limma” (linear models for microarray data) R package [28] to detect all DEGs between PM/DM and healthy controls. The values for statistical significance were set as adjusted *P*≤0.05 and |log_2_ fold change (FC)|≥1, except for GSE39454 PM and GSE39454 DM, which were set as *P*≤0.05 and |log_2_FC|≥1. Volcano maps were drawn using the “ggplot2” [29] package. Principal component analysis (PCA) was used to extract two features from each group of genes. PCA score trajectory plots can be used to show whether there is overlap between PM/DM and the control group. When there was no substantial overlap, it suggested that there was a significant difference between PM/DM and the control group, which could be analyzed in the next step.

### RRA analysis

To reduce the discrepancies and merge the outcomes of multiple microarray studies, we conducted RRA analysis to recognize substantial DEGs. RRA is an efficient tool to combine results from several arrays [30]. First, we acquired lists of up-ranked and down-ranked genes of each series that were produced by analyzing the FC of expression between PM/DM and controls. The “Robust Rank Aggregation” R package was utilized to aggregate all sorted gene lists in the datasets [30]. The Benjamini and Hochberg false discovery rate (FDR) method was utilized to generate the adjusted *P*-value ranked genes with adjusted *P*<0.05 and log_2_FC>0.5 were regarded as significant (set 1).

### Functional and pathway enrichment analysis

To examine the effect of DEGs on the pathogenesis of PM/DM, we executed GO functional enrichment analysis and KEGG pathway analysis of the significant genes identified by RRA. The “clusterProfiler” R package automates the classification of biological terminology and gene cluster enrichment analysis. The analysis and visualization modules were amalgamated into a repeated workflow [31]. Furthermore, we used the “GOplot” R package (circle plot, chord plot, cluster plot) to append quantitative data about pathways to the GO terms of interest [32]. An adjusted *P*<0.05 and FDR <0.05 were considered the criteria for judgment.

### Enrichment analysis by GSEA and GSVA

To identify the possible functional pathways associated with DEGs in PM/DM, we executed GSEA to investigate the biological processes and pathways of these genes and used the GSEA plot function of the “clusterProfiler” R package to carry out GSEA [31]. The annotated gene set “h.all.v7.0.symbols.gmt” in the MsigDB V6.2 database [33] was regarded as a reference gene set. FDR<0.25, normalized *P*<0.05 and |normalized enrichment score (NES)|>1 were deemed to show meaningful enrichment. Further, we used the gseaplot2 function of the “clusterProfiler” R package to visualize the results of GSEA [31]. The R package “GSVA” was utilized to calculate the signaling pathways in enrolled datasets [34]. Subsequently, the R package “limma” was used to research the meaningful distinguishing gene sets between PM/DM and healthy controls [28]. The gene set “h.all.v7.0.symbols.gmt” was chosen as the reference gene set. Volcano maps were drawn using the “ggplot2” [29] package. The “pheatmap” package was used to generate the heatmap plots to visualize the results of GSVA analysis [35]. Signaling pathways that were usually enriched by GSEA and GSVA analysis were regarded as possible PM/DM-related pathways.

### WGCNA

We used the “WGCNA” R package to make a co-expression network on the basis of the above DEGs. We built a weighted adjoining matrix, identified outliers by sample clustering, and removed the outliers. In addition, we defined the association degree (soft threshold parameter) to indicate a strong intergenic association while carrying out scale-free network verification. Further, we converted the adjoining matrix to a topological overlap matrix (TOM) to estimate the degree of connectedness between genes. To avoid bias and error, the minimum number of genes in each module was set to 30. Using the average linkage hierarchical clustering method on account of the TOM discrepancy measure, we categorized the genes with similar expression spectra using gene modules, displaying them on branches and with different colors of clustering trees to display the relationships between modules. The correlation cutoff was set at 0.8. To verify the interactions occurring significantly above the expected probability due to chance, a control network analysis was also performed using a Z-test, and q-value analysis was also performed to the correction analysis of multiple tests. The relationship between gene module and phenotype was computed and the modules related to clinical characteristics were selected.

### Assessment of immune cell infiltration by CIBERSORT

Here, we adopted the CIBERSORT method to analyze the expression of 22 immune cell categories across seven gene expression matrix datasets [36]. We selected the characteristic gene matrix file LM22, which is a leukocyte gene characteristic matrix for identifying 22 human hemopoietic cell phenotypes [36]. We then set the run mode to batch mode, utilized the quantile standardization of expression matrix, and set 500 permutations for penetration analysis. Results with *P*<0.05 were considered statistically significant. The Mann-Whitney U test was utilized to discover the meaningfully distinct infiltrating immune cell sorts between PM/DM and healthy controls. In addition, we used “ggplot2” package [29] to design boxplots to show the differentiation in immune cell infiltration.

### PPI network analysis

The STRING database is an online database for retrieving known proteins and forecasting PPI, including the direct physical interrelations between proteins and their connections to indirect functions [37]. We provided the meaningful genes from the above-mentioned RRA analysis to the STRING database to construct a PPI network (set 2). We set the medium confidence score as>0.4 when outputting the network analysis results and exported the TSV format data to Cytoscape3.7.2 [38]. The CytoHubba (version 0.1) plug-in for Cytoscape was utilized to distinguish the association degrees of genes in the PPI network and visualize the network. The Molecular Complex Detection (MCODE version 1.6.1) software (http://apps.cytoscape.org/apps/mcode) was adopted to choose the key modules from the PPI network in Cytoscape with MCODE scores >5 (set 3 and set 4).

### Venn diagram analysis

Venn diagram analysis was carried out using the Venn Diagram R package (version 2.12.1) [39] for the DEGs of seven datasets. Overlapping DEGs (set 5) were maintained for further analysis. The comprehensive analysis of sets 1- 5 indicated the core genes. The core genes were used for the next analysis.

### Correlation between core genes and muscle injury

We searched for muscle injury-associated genes using the GeneCards website (https://www.genecards.org/) with the term “muscle injury.” A relevance score based on a scale of 0 to 100 was used to demonstrate the strength of the association between genes and muscle injury. The higher the score, the more relevant it was. The relevant scores were sorted in descending order, and the top 16 genes were considered the uppermost muscle injury-related genes. Furthermore, we used the “corrplot” package [40] to calculate the correlation between core genes and muscle injury-related genes. In addition, we used “ggcorrplot” package [41] to visualize the correlation. The final core genes were determined according to the correlation score.

### Diagnostic effectiveness evaluation

For diagnostic analysis, we selected GSE39454 and GSE128470, which contain both PM and DM samples. In addition, we carried out confirmation studies using data from a microarray dataset (GSE48280, including 5 DM patients, 5 PM patients, and five healthy controls) [42]. The receiver operator characteristic (ROC) curves were diagramed and area under curve (AUC) was counted respectively to appraise the performance of each dataset (GSE39454 PM, GSE39454 DM, GSE128470 PM, GSE128470 DM, GSE48280 PM and GSE48280 DM) utilizing the R packages “pROC” [43]. We defined a guideline to distinguish different diagnostic criteria, including excellent accuracy (0.9≤AUC<1), decent accuracy (0.8≤AUC<0.9), fair accuracy (0.7≤AUC<0.8), poor accuracy (0.6≤AUC<0.7), and insufficient accuracy (0.5≤AUC<0.6) [44]. If the AUC value of a hub gene was >0.8, it was regarded as having excellent specificity and sensitivity for identifying PM/DM.

## RESULTS

### Essential information of selected microarrays

**Figure 1:**
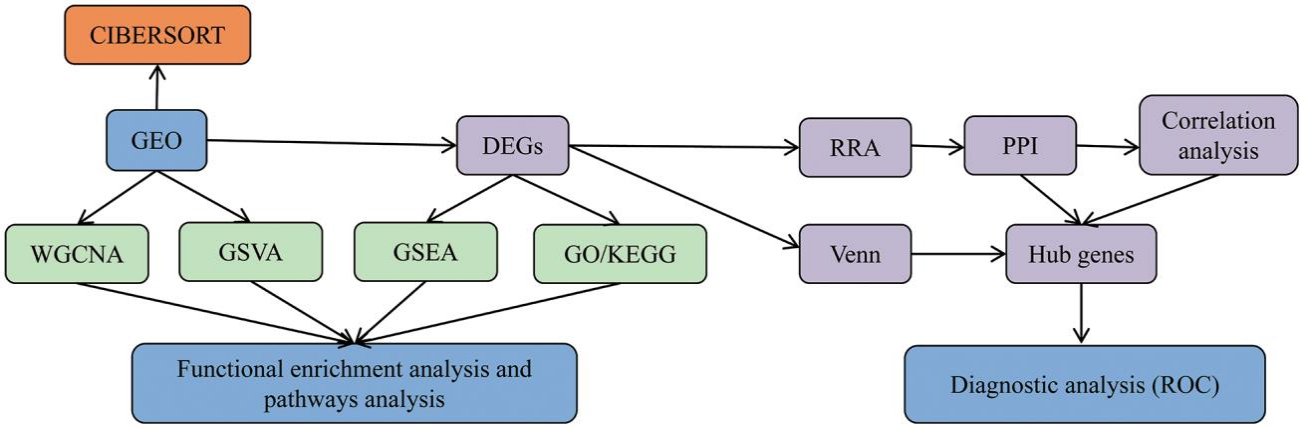
**Data analysis and processing flow.** The data processing in this study is divided into five steps.

In light of the above inclusion criteria, GSE1551, GSE3112, GSE39454, GSE46239, and GSE128470 were finally selected. A total of 145 samples (81 DM samples, 22 PM samples, and 42 control samples) were evaluated in our study. Analyses of GSE1551, GSE3112, and GSE128470 series were undertaken on the GPL96 platform (Affymetrix Human Genome U133A Array), and GSE39454 and GSE46239 were performed on the GPL570 platform (Affymetrix Human Genome U133 Plus 2.0 Array). The details of these datasets are presented in Table 1. Our analysis of microarray data is based on the basic workflow (Figure 1).

**Table 1:** Characteristics of the enrolled microarray datasets.

**GSE ID**	**DM**	**PM**	**Control**	**Tissues**	**Analysis type**	**Platform**	**Year**
GSE1551	13		10	Muscle	Array	GPL96	2005
GSE3112		6	11	Muscle	Array	GPL96	2005
GSE39454	8	9	5	Skeletal muscle	Array	GPL570	2012
GSE46239	48		4	Skin	Array	GPL570	2013
GSE128470	12	7	12	Muscle	Array	GPL96	2019

### Evaluation of DEGs in PM/DM

**Figure 2:**
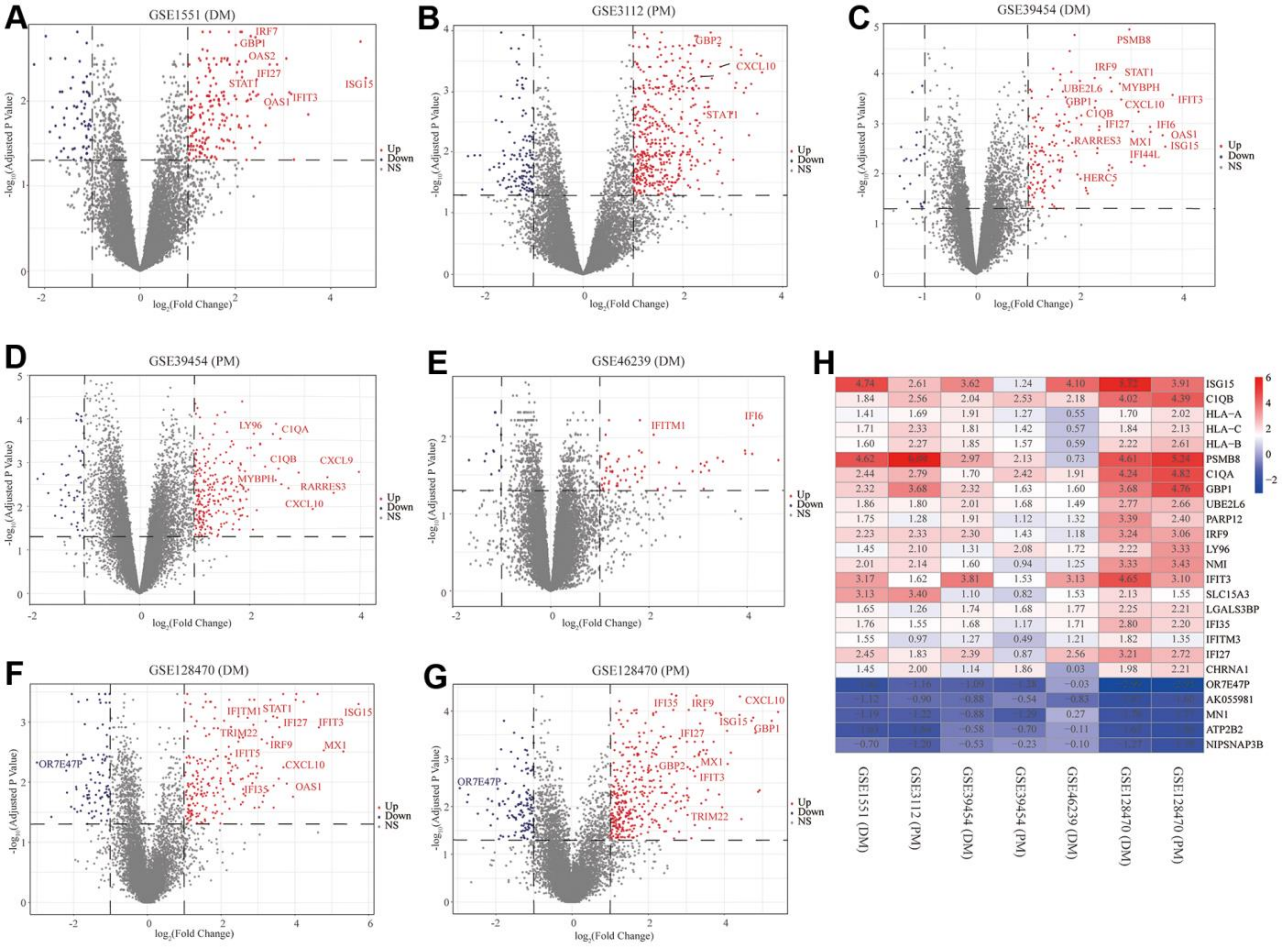
**Volcano diagrams of the seven microarrays and heatmap of the robust rank aggregation (RRA) analysis.** Red points indicate upregulated genes, while blue points indicate downregulated genes. Gray points indicate genes with no meaningful difference. (**A**) GSE1551 (dermatomyositis, DM); (**B**) GSE3112 (polymyositis, PM); (**C**) GSE39454 (DM); (**D**) GSE39454 (PM); (**E**) GSE46239 (DM); (**F**) GSE128470 (DM); (**G**) GSE128470 (PM); (**H**) Heatmap of the top 20 upregulated and five downregulated genes in the RRA method. Red and blue indicate high and low expression of genes in patients with PM/DM, respectively.

First, to exclude individual variations between participants, all five microarray datasets were normalized using the “affy” R package. The standardized boxplots are presented in Supplementary Figure 1; all participants in each dataset attained acceptable homogeneity. Secondly, the PCA plots of all data series are shown in Supplementary Figure 2. In view of the gene expression of all participants, PCA showed differing distribution patterns between PM/DM and control groups. The ranges between the participants in the control group were similar, as were the ranges between the participants in the DM or PM groups. Furthermore, we utilized the “limma” R package to screen out the DEGs on account of the above screening criteria, and the volcano plots of the seven microarrays are displayed in Figure 2.

### RRA integrated analysis of DEGs

The RRA analysis posits that each gene is irregularly arranged in each dataset. The lower the *P*-value in RRA outcomes, the higher the gene grade and the reliability of distinguishing gene expression. After the integrated analysis, 82 significant DEGs (70 upregulated and 12 downregulated) were identified (Supplementary Table 1; set 1). The heatmap of the top 20 upregulated and five downregulated genes is displayed in Figure 2H. The top 10 meaningfully upregulated genes found in PM/DM included *ISG15* (*P* = 4.77E-08), *C1QB* (*P* = 7.73E-08), *HLA-A* (*P* = 9.33E-07), *HLA-C* (*P* = 1.53E-06), *HLA-B* (*P* = 2.15E-06), *PSMB8* (*P* = 7.57E-06), *C1QA* (*P* = 9.28E-06), *GBP1* (*P* = 1.05E-05), *UBE2L6* (*P* = 1.07E-05), *PARP12* (*P* = 3.04E-05).

### Functional annotation

**Figure 3:**
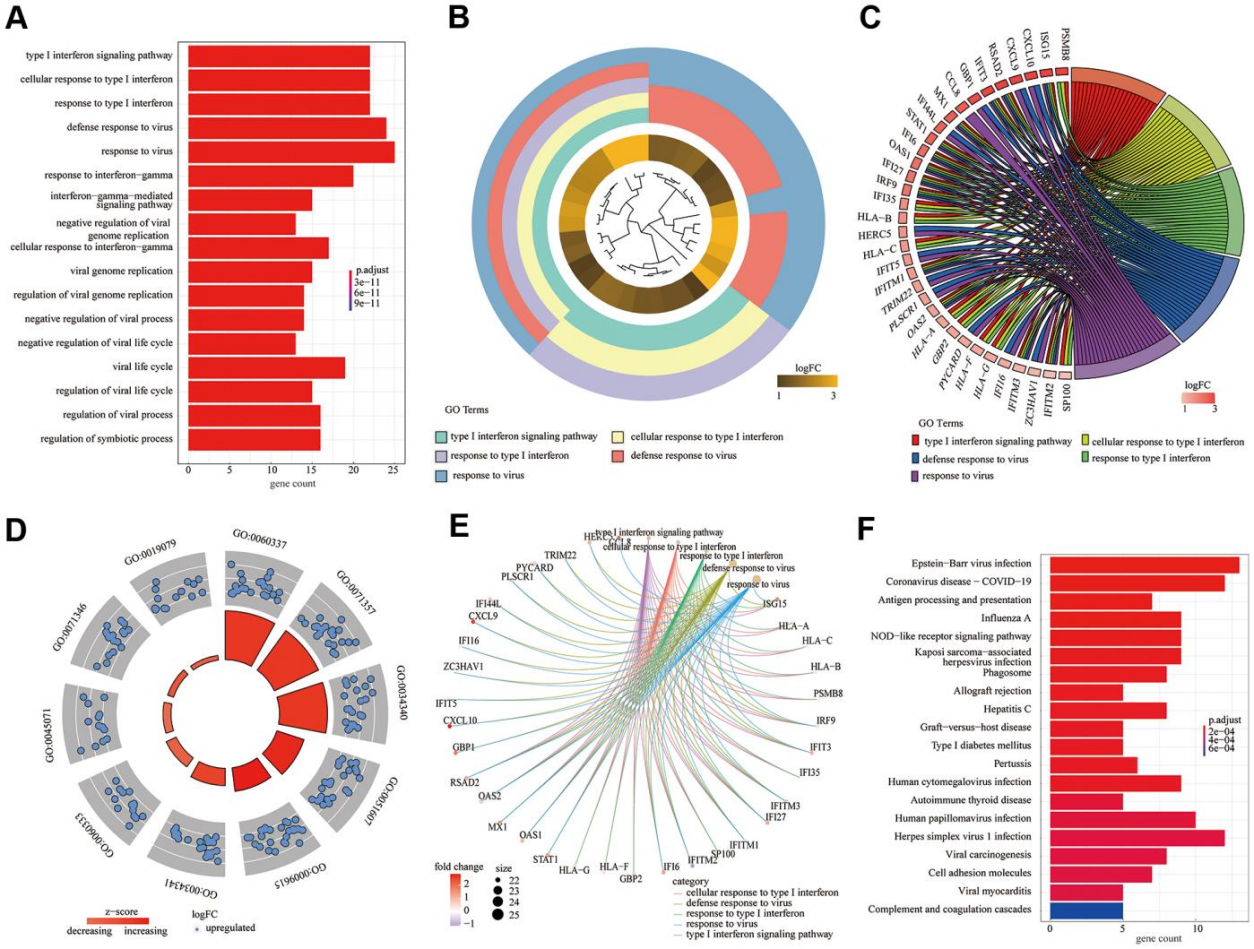
**Gene ontology (GO) functional enrichment analysis and Kyoto encyclopedia of genes and genomes (KEGG) pathway analysis of differentially expressed genes.** (**A**) GO bar plot (Figure 3A); (**B**) GO cluster plot; (**C**) GO chord plot; (**D**) GO circle plot; (**E**) GO cneplot; (**F**) KEGG bar plot.

According to the above experimental results, 82 DEGs were subjected to GO [including three main function modules: biological process (BP), molecular function (MF), and cellular component (CC)] and KEGG analyses. The analyses demonstrated that the type I interferon signaling pathway (GO: 0060337; adjusted *P* = 2.14E-31) was the most meaningfully enriched BF, followed by cellular response to type I interferon (GO: 0071357; adjusted *P* = 2.14E-31) and response to type I interferon (GO: 0034340; adjusted *P* = 3.93E-31). Different analysis methods showed differing results; thus, we used the GO bar plot (Figure 3A), cluster plot (Figure 3B), chord plot (Figure 2C), circle plot (Figure 3D), and cneplot (Figure 3E) to visualize the DEGs and GO terms. Furthermore, KEGG pathway enrichment analysis showed that Epstein-Barr virus infection (hsa05169; adjusted *P* = 7.91E-09) and coronavirus disease - COVID-19 (hsa05171; adjusted *P* = 2.98E-07) were significantly enriched (Figure 3F).

### GSEA and GSVA

**Figure 4:**
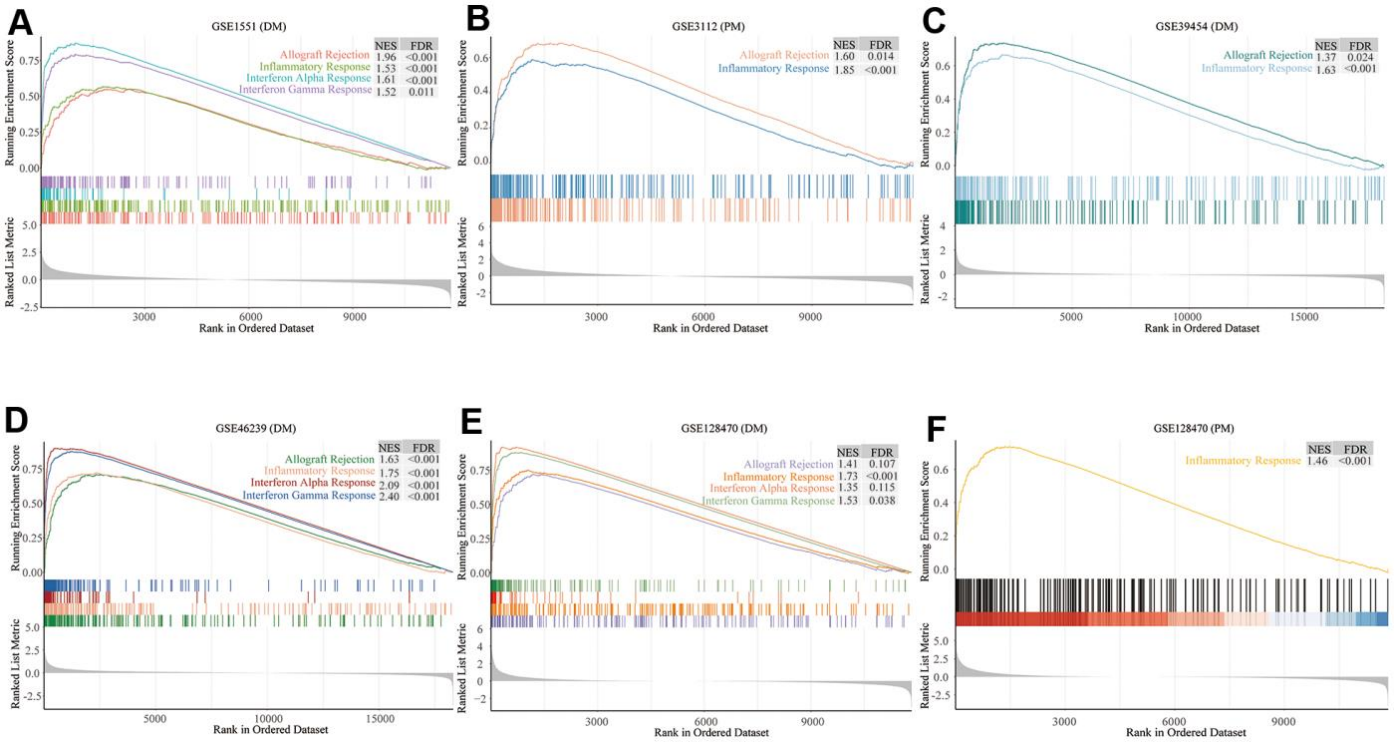
**Gene set enrichment analysis (GSEA) results of six microarrays.** Normalized enrichment score (NES) demonstrates the analysis outcomes across gene sets. False discovery rate (FDR) indicates if a set was meaningfully enriched. (**A**) GSE1551 (dermatomyositis, DM); (**B**) GSE3112 (polymyositis, PM); (**C**) GSE39454 (DM); (**D**) GSE46239 (DM); (**E**) GSE128470 (DM); (**F**) GSE128470 (PM).

**Figure 5:**
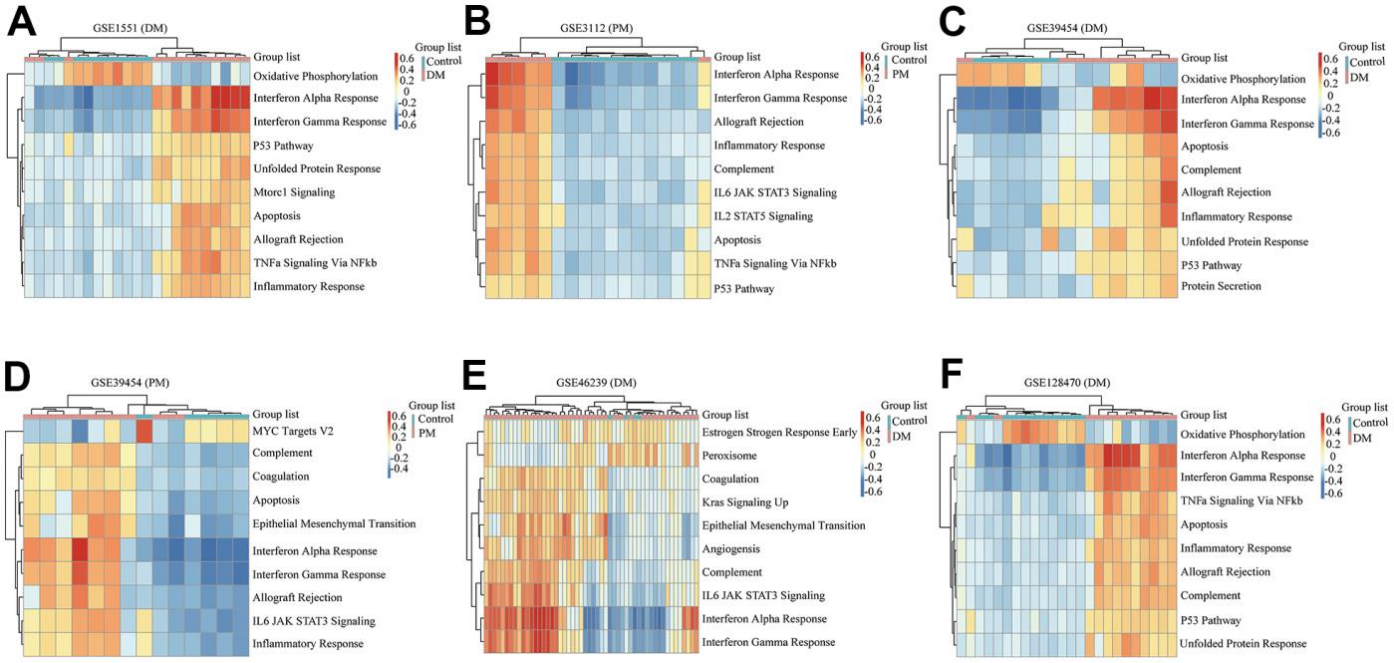
**Gene set variation analysis (GSVA) results of six microarrays.** (**A**) GSE1551 (dermatomyositis, DM); (**B**) GSE3112 (polymyositis, PM); (**C**) GSE39454 (DM); (**D**) GSE39454 (PM); (**E**) GSE46239 (DM); (**F**) GSE128470 (DM).

**Figure 6:**
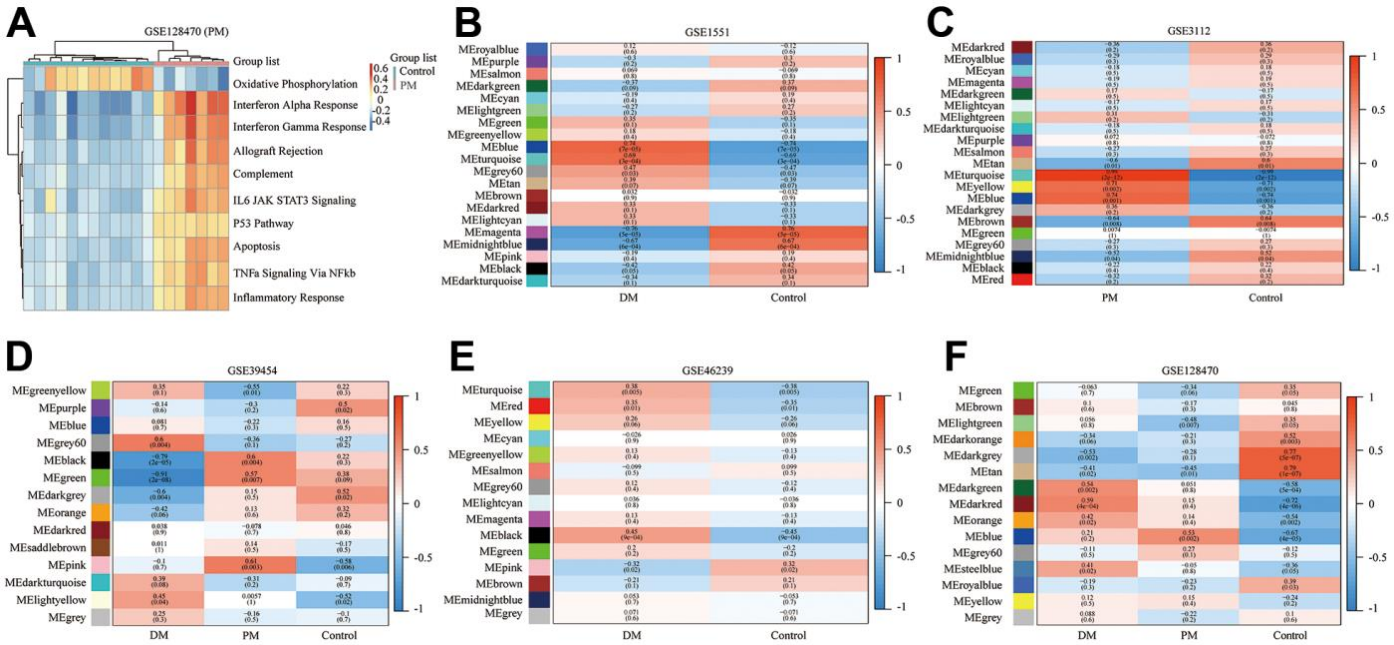
**Gene set variation analysis (GSVA) of GSE128470 (polymyositis, PM) and weighted gene co-expression network analysis (WGCNA) results of five datasets.** (**A**) GSVA result of GSE128470 (PM); (**B**) WGCNA result of GSE1551; (**C**) WGCNA result of GSE3112; (**D**) WGCNA result of GSE39454; (**E**) WGCNA result of GSE46239; (**F**) WGCNA result of GSE128470.

We comprehensively analyzed the results of all GSEA and GSVA datasets. We screened out four commonly enriched pathways: allograft rejection, inflammatory response, interferon-alpha response, and interferon-gamma response (Figures 4–6A and Supplementary Figure 3). Analyses of the present research revealed that interferon response and inflammatory response were the biological pathways most relevant to the pathogenesis of PM/DM.

### WGCNA

In this study, the WGCNA R package was utilized for co-expression network construction. Before analysis, we determined suitable soft thresholds to establish a scale-free network (Supplementary Figure 4). To ensure the scale-free topology model curve fit the plateau smoothly, different soft thresholds were selected for each dataset (Supplementary Figure 4). Hereafter, the stepwise method was used for dynamic cluster analysis. First, dynamic hybrid cutting was used to generate the dendrogram (Supplementary Figure 5). Each leaf represents a single gene that has a close expression profile when placed together to form a branch, and the branch represented a gene module. Subsequently, we also computed the characteristic genes of each module and clustered the modules in parallel, especially when the correlation was >0.8; then, these modules were merged. Thus, we obtained significant modules excluding the nonsense one (gray; Supplementary Figure 5). The adjacency heatmap of characteristic genes showed that the red and blue modules were the most positively and negatively correlated with the occurrence of PM/DM, respectively (Figure 6B–6F). Taken together, these results indicated that MEblue and MEturquoise are key target modules in PM/DM.

### Immune cell infiltration outcomes

**Figure 7:**
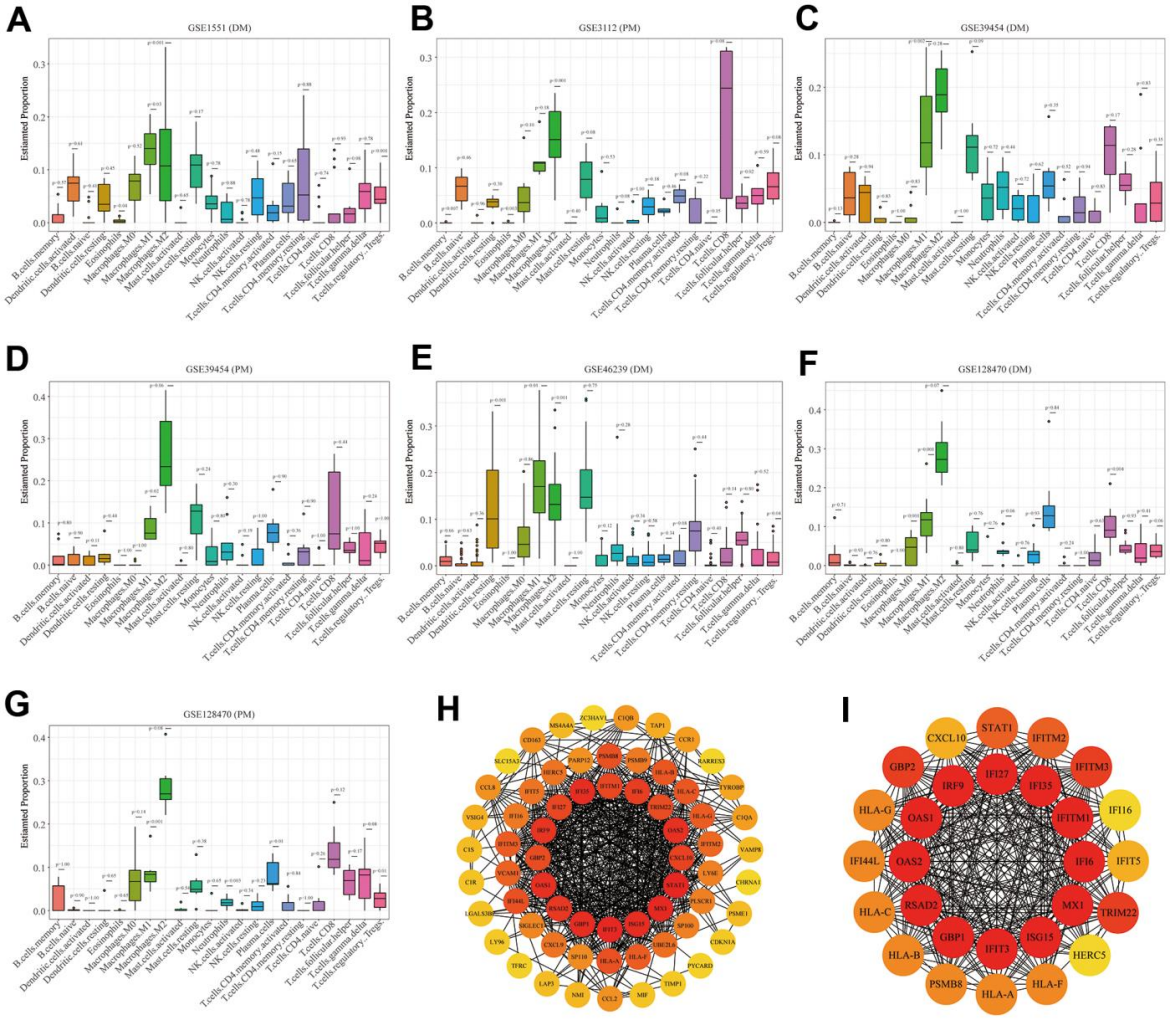
**Boxplots of the proportion of 22 immune cell sorts in polymyositis (PM) and dermatomyositis (DM) and the outcomes of protein–protein interaction (PPI) network analysis.** (**A**) GSE1551 (DM); (**B**) GSE3112 (PM); (**C**) GSE39454 (DM); (**D**) GSE39454 (PM); (**E**) GSE46239 (DM); (**F**) GSE128470 (DM); (**G**) GSE128470 (PM); (**H**) Results of Cytoscape plug-in CytoHubba; (**I**) the top significant module of Cytoscape plug-in MCODE.

The boxplots of the immune cell infiltration results demonstrated that compared with those in the healthy control participants (immune cell infiltration results of healthy controls are shown in Supplementary Figure 6), M1 (*P* = 0.03) and M2 (*P*<0.0001) macrophage infiltration in GSE1551 was higher and that of regulatory T cells (Tregs) (*P*< 0.0001) in GSE1551 was less (Figure 7A). M2 macrophages (*P*< 0.0001) in GSE3112 infiltrated more, and memory B cells (*P* = 0.007), eosinophils (*P* = 0.003) and follicular helper T cells (*P* = 0.015) in GSE3112 infiltrated less (Figure 7B). M1 macrophages (*P* = 0.002) in GSE39454 (DM) infiltrated more (Figure 7C), and M1 macrophages (*P* = 0.019) in GSE39454 (PM) infiltrated more (Figure 7D). M1 macrophages (*P* = 0.01) and M2 macrophages (*P* = 0.001) in GSE46239 infiltrated more and resting dendritic cells (*P* = 0.001) and Tregs (*P* = 0.041) in GSE46239 infiltrated less (Figure 7E). M0 macrophages (*P*< 0.0001) and M1 macrophages (*P* = 0.001) in GSE128470 (DM) infiltrated more, and CD8 T cells (*P* = 0.004) in GSE46239 infiltrated less (Figure 7F). M1 macrophages (*P* = 0.001) in GSE128470 (PM) infiltrated more, and neutrophils (*P* = 0.003), plasma cells (*P* = 0.01) and Tregs (*P* = 0.013) in GSE128470 (PM) infiltrated less (Figure 7G). Overall, our results indicated that M0, M1, and M2 macrophages in PM/DM patients infiltrated more, and Tregs in PM/DM patients infiltrated less.

### PPI network analysis

**Figure 8:**
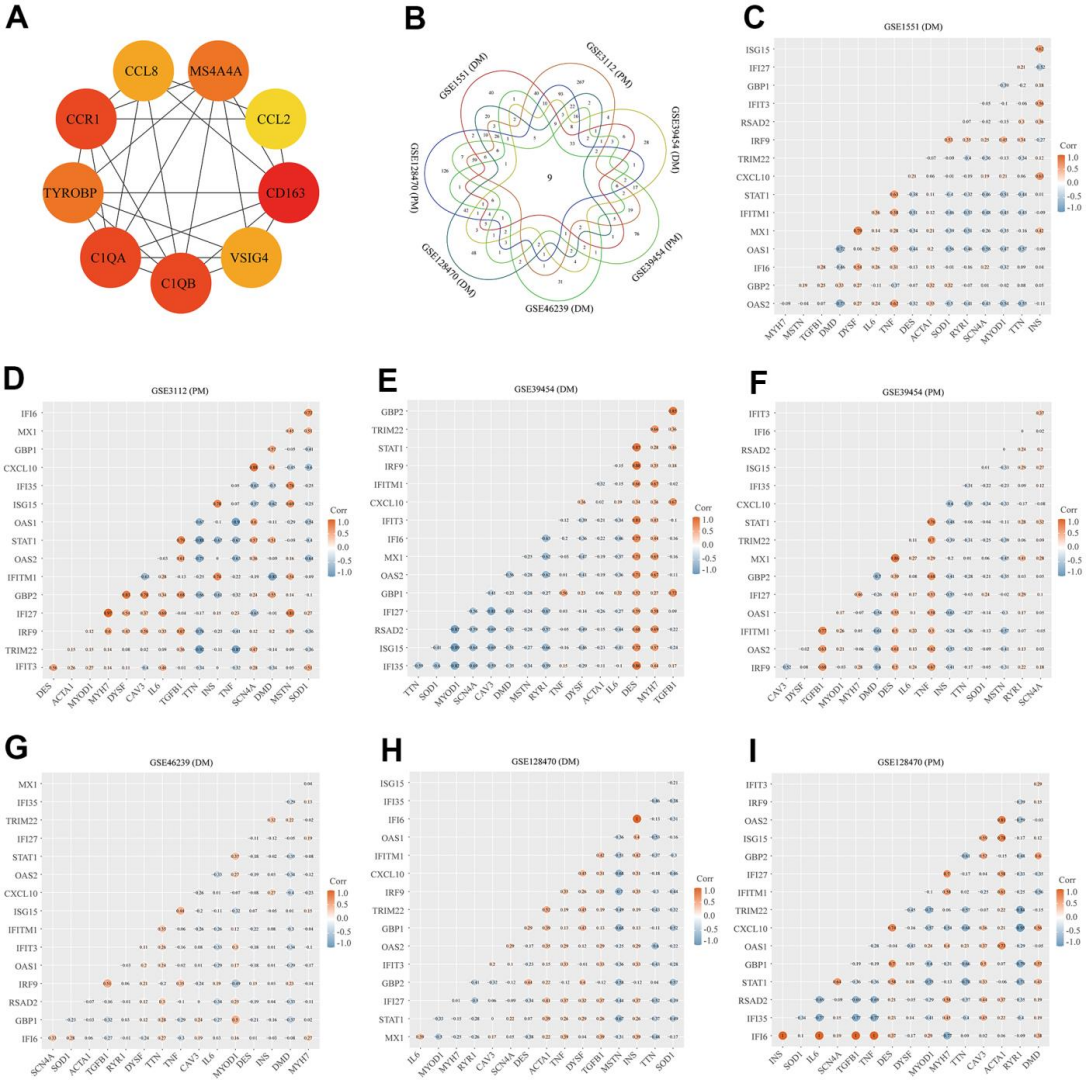
**Protein–protein interaction (PPI) network analysis, Venn diagram analysis and correlation between core genes and muscle injury.** (**A**) The top two significant modules of Cytoscape plug-in MCODE. (**B**) Results of Venn diagram analysis; (**C**) Association result of GSE1551 (DM); (**D**) Association result of GSE3112 (PM); (**E**) Association result of GSE39454 (DM); (**F**) Association result of GSE39454 (PM); (**G**) Association result of GSE46239 (DM); (**H**) Association result of GSE128470 (DM); (**I**) Association result of GSE128470 (PM).

Using the STRING online database, we input significant genes from the RRA method for PPI network analysis, and we used Cytoscape software to show the results. In the PPI network, the connectivity between nodes was highlighted to determine the interrelations between the proteins encoded by genes in PM/DM, and a PPI network of 65 object genes was established. The genes situated in the centric node were recognized as key genes that may play crucial physiological regulatory actions in PM/DM (Figure 7H, orange node genes) (set 2). Among the 65 nodes, 16 proteins were picked by degree through Cytoscape CytoHubba plug-in; namely, central genes (*ISG15*, *MX1*, *STAT1*, *CXCL10*, *OAS2*, *TRIM22*, *IFI6*, *IFITM1*, *IFI35*, *IFI27*, *IRF9*, *GBP2*, *OAS1*, *RSAD2*, *GBP1*, and *IFIT3*) (Figure 7H, orange node genes) (set 2). In addition, another Cytoscape plug-in, MCODE, was used to further categorize the PPI to recognize sub-network. Here, we utilized MCODE for cluster analysis. The top meaningful module was selected after clustering (Figure 7I, orange node genes) (set 3). Cluster 1 included 28 genes and the 12 core genes in the central node contained *ISG15*, *MX1*, *IFI6*, *IFITM1*, *IFI35*, *IFI27*, *IRF9*, *OAS1*, *OAS2*, *RSAD2*, *GBP1*, and *IFIT3*. The top two significant module was obtained and included nine genes (*C1QB*, *VSIG4*, *CD163*, *CCL2*, *MS4A4A*, *CCL8*, *CCR1*, *TYROBP*, and *C1QA*) (Figure 8A) (set 4).

### Venn diagram analysis and identification of core genes

We used the Venn diagram analysis for DEGs of seven datasets and identified nine overlapping genes (Figure 8B) (set 5), including *ISG15*, *IFIT3*, *IRF9*, *LY96*, *C1R*, *CD163*, *C1QB*, *C1QA*, and *LGALS3BP* (Figure 8B). The comprehensive analysis of set 1, set 2, set 3, set 4, and set 5 recognized core genes. The core genes, including, *IFIT3*, *ISG15*, *MX1*, *STAT1*, *CXCL10*, *OAS2*, *TRIM22*, *IFI6*, *IFITM1*, *IFI35*, *IFI27*, *IRF9*, *GBP2*, *OAS1*, *RSAD2*, and *GBP1* were selected for further analysis.

### Correlation between core genes and muscle injury

We searched genes associated with the muscle injury in the GeneCards database. The muscle injury-related genes in the top 16 of relevance scores contained *RYR1*, *TTN*, *DMD*, *IL6*, *TNF*, *CAV3*, *DES*, *MSTN*, *DYSF*, *INS*, *MYOD1*, *ACTA1*, *MYH7*, *TGFB1*, *SOD1*, and *SCN4A*. The correlation between muscle injury-related genes in the top 16 of relevance scores and above core genes was analyzed (Figure 8). According to the results of correlation analysis, we identified five final hub genes (*TRIM22*, *IFI6*, *IFITM1*, *IFI35*, and *IRF9*) associated with PM/DM, which were used for subsequent investigation. Hub genes are generally regarded as key functional genes and are highly associated with other genes.

### Diagnostic value of the five hub genes

**Figure 9:**
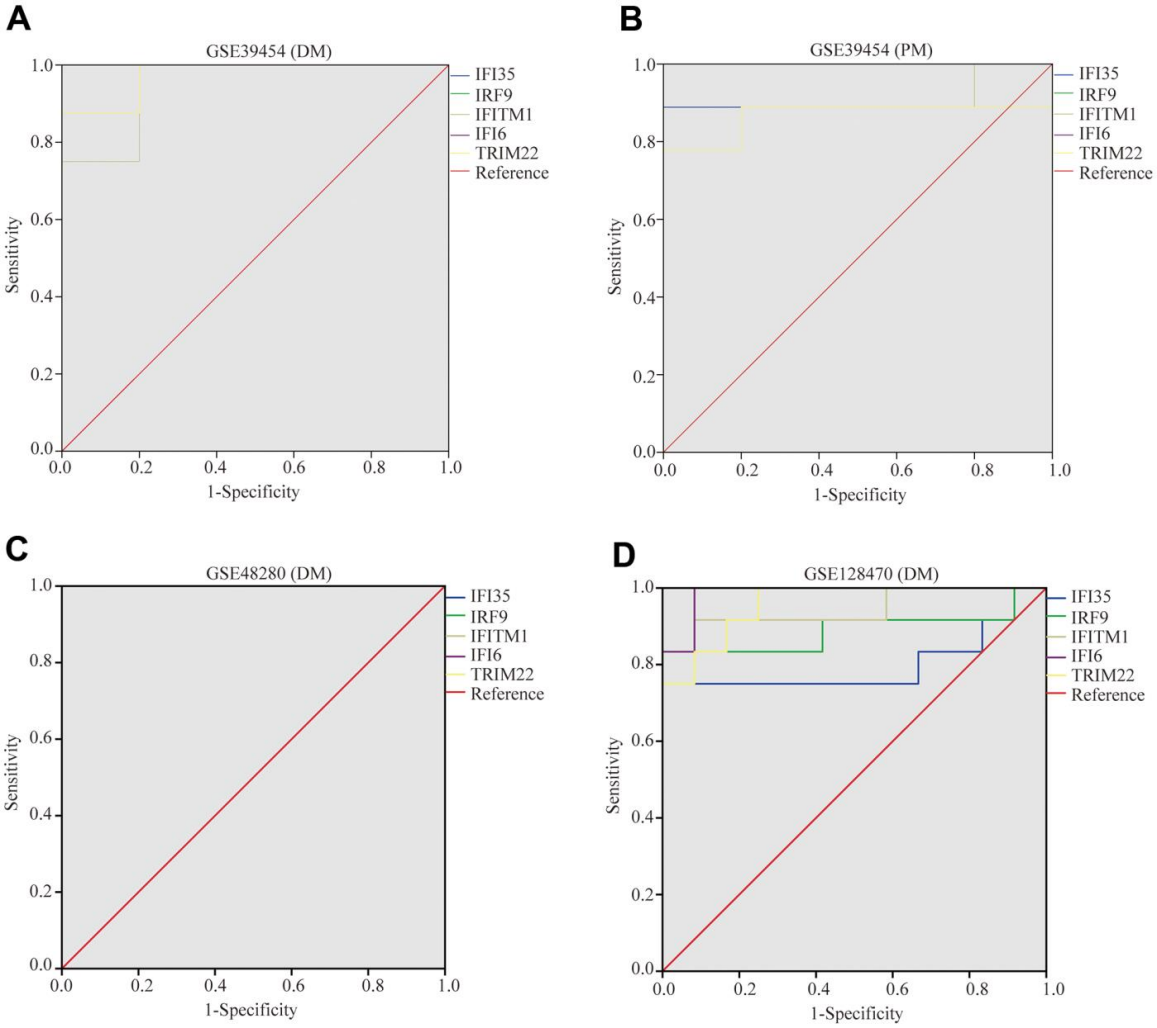
**Receiver operating characteristics of five hub genes.** (**A**) GSE39454 (DM); (**B**) GSE39454 (PM); (**C**) GSE48280 (DM); (**D**) GSE128470 (DM).

To validate the diagnostic value of five hub genes (*TRIM22*, *IFI6*, *IFITM1*, *IFI35*, and *IRF9*) in PM/DM patients, the next phase of our study was to execute ROC analyses to investigate the sensitivity and specificity of hub genes for PM/DM diagnosis. The ROC outcomes verified that five hub genes could differentiate between PM/DM patients and healthy controls in GSE39454 DM, GSE39454 PM, GSE48280 DM and GSE128470 DM (all *P*<0.05), and the AUCs were between 0.799 and 1 (Figure 9A–9D). However, the diagnostic value of the five hub genes (especially *IFI35* and *IFI6*) in GSE1551DM, GSE3112PM, and GSE46239 DM was uncertain (Supplementary Figure 7A–7E). This result may be attributed to the large difference in sample size between DM patients and healthy controls in GSE46239 and the small sample sizes of GSE1551 and GSE3112 that lead to some bias. Our results indicated that expression of *TRIM22*, *IFI6*, *IFITM1*, *IFI35*, and *IRF9* was related to disease diagnosis and that these genes could act as biomarkers to verify the diagnosis of PM/DM and validate the effectiveness of PM/DM treatment.

## DISCUSSION

PM and DM are common autoimmune disorders clinically presenting with skeletal muscle injury [45]. At present, there is no specific diagnostic antibody for PM and DM. The diagnosis mainly depends on medical experience and invasive muscle biopsy [45]. Hence, there is an imperative need to better understand the pathogenesis of PM/DM to generate novel strategies for the diagnosis and treatment of PM/DM.

In the current research, a large number of bioinformatic analysis tools have been used to identify five hub genes (*TRIM22*, *IFI6*, *IFITM1*, *IFI35*, and *IRF9*) between PM/DM and health control subjects on the basis of gene expression profiles attained from GSE1551 (DM), GSE3112 (PM), GSE39454 (DM), GSE39454 (PM), GSE46239 (DM), GSE128470 (DM), and GSE128470 (PM) datasets. We explored the biological functions of these DEGs utilizing GO enrichment and KEGG pathway analyses; these results demonstrated that DEGs are significantly associated with the IFN response pathway. Moreover, deep enrichment analysis by GSEA and GSVA revealed that immune function and the IFN signaling pathway are pivotal features implicated in PM/DM, which was in accordance with the findings of previous studies [13–17, 46]. Most notably, WGCNA was conducted to identify meaningful modules correlated with PM/DM, indicating that MEblue and MEturquoise could be identified as key target modules in PM/DM. Furthermore, this study is the first to use a bioinformatics analysis with an immune cell infiltration analysis in PM/DM. The results indicated that M0 macrophages, M1 macrophages, and M2 macrophages in PM/DM patients infiltrated more, and Tregs in PM/DM patients infiltrated less. We performed PPI network analysis and Venn diagram analysis, and we assessed the associations between core genes and muscle injury to identify hub genes. Next, we performed ROC analyses to investigate the sensitivity and specificity of five hub genes for the diagnosis of PM/DM, and results indicated that expression of *TRIM22*, *IFI6*, *IFITM1*, *IFI35*, and *IRF9* are related to the diagnosis of PM/DM.

The five hub genes (*TRIM22*, *IFI6*, *IFITM1*, *IFI35*, and *IRF9*) are all IFN pathway genes. In all signaling pathways of PM/DM, the one centered on IFNs has been the most studied and IFNs have been identified as playing vital roles in PM/DM. Initial investigation of cytokine expression revealed the upregulation of IFN-γ in PM/DM muscle [47], which leads to the localized overexpression of IFN-γ-related genes [48]. These findings suggested that transcriptomic changes are involved in the pathogenesis of PM/DM. According to the cell surface receptor binding ligand family, the IFN pathway is divided into three categories: type 1 IFNs (IFN1; including IFN-α and IFN-β), type 2 IFNs (IFN2 or IFN-γ), and type 3 IFNs (IFN3 or IFN-λ), which share overlapping signaling pathways [49, 50]. Analysis has suggested that IFN1 is potentially relevant to the pathogenesis of DM [13, 14]. In particular, overexpression of IFN1-related genes has been described in the muscle [13], skin [14], and circulating leukocytes [15, 16] of DM patients. Furthermore, the expression of IFN1-induced genes is associated with the disease activity of DM [15, 16]. In addition, a previous study on the skin of DM patients demonstrated that both IFN-β and IFN-γ are highly correlated with the degree of the IFN transcriptional response, whereas IFN-α are not [14]. However, related investigation on the IFN pathway in the pathogenesis of PM is lacking. Although both PM and DM were implicated in some studies, the IFN pathway was found to be associated with DM but not with PM [13, 17]. Nevertheless, in other studies, PM and DM samples showed similar gene expression profiles, although there were differences in several other details [15, 46]. Thus, the role of the IFN pathway in the pathogenesis of PM/DM remains controversial.

*TRIM22* encodes an IFN-induced member of the tripartite motif (TRIM) family, which is localized to the cytoplasm. Previous studies indicated that this protein might participate in the antiviral effects of IFN [51]. Previous reports on TRIMs in autoimmunity have been inconclusive. TRIMs, as active modulators of IFN production and inflammasome activity, may counteract autoimmune or inflammatory disorders if their activity or expression is improved. Conversely, TRIMs that negatively regulate IFN production or inflammasome activity may be beneficial in some diseases, as they can prevent the excessive production of pathogenic cytokines, thus avoiding the progression of autoimmune and autoinflammatory diseases [52]. In addition, the researchers found that variants in *TRIM22* influenced the activation of NOD2-dependent IFN-β signaling and the upregulation of NF-κB pathways in early-onset inflammatory bowel disease, and the TRIM22-NOD2 network affected antiviral pathways leading to inflammation [53]. Meanwhile, only one early-onset Crohn’s disease (CD) patient had a *TRIM22* variant that was consistent with the previous result [54]. These results suggested the *TRIM22* variant affects pediatric patients with inflammatory bowel disease. Another study showed that psoriasis was associated with the overexpression of antiviral genes (such as *ISG15* and *TRIM22*) in the skin, but not in the blood [55]. However, the role of *TRIM22* in the development of multiple sclerosis (MS) has been debatable [56–58]. Therefore, the role of *TRIM22* in connective tissue disorders needs further elucidation and interpretation.

*IFI6*, also known as *G1P3*, is one of the IFN-stimulating genes (ISGs). IFI6 may be crucial in mediating apoptosis in many cancers [59], and it may have antiviral activity against the hepatitis C virus [60]. Furthermore, the expression of IFI6 is elevated in the blood or platelet samples of patients with systemic lupus erythematosus (SLE), rheumatoid arthritis (RA), primary antiphospholipid syndrome, and MS [61–63]. Meanwhile, bioinformatic analysis of psoriasis and SLE cases suggested *IFI6* is a primary IFN-inducible gene [64, 65]. *IFITM1*, a member of the IFN-induced transmembrane protein family, is a known regulator of immunity and antiviral activity [66]. It is interesting to note that mounting evidence has shown that IFITM1 is highly expressed in many tumor tissues [67]. However, the definite mechanism of IFITM1 in autoimmune diseases remains unclear. *IFI35* encodes IFN-induced 35 kDa protein, an IFN-induced protein, which mainly participates in, and regulates the response of, the innate immune system [68–70]. IFI35 forms complexes with N-myc and STAT interactor (NMI), regulating various immune responses, such as restricting virus-triggered IFN-β production [70] and passively adjusting NF-κB signaling, leading to the restraint of endothelial cell proliferation and migration [70]. IFI35 plays different roles in connective tissue disease. For example, IFI35 is deemed a biomarker of neuroinflammation and therapeutic reaction in MS [71]. Additionally, IFI35 promotes the proliferation of mesangial cells, which is modulated by methyl CpG-binding domains in lupus nephritis [72].

*IRF9* encodes a transcription factor that plays a principal role in antiviral immunity. It participates in the IFN response and modulates cell proliferation [73] and immune system activity [74–76]. In general, *TRIM22, IFI6, IFITM1, IFI35*, and *IRF9* are IFN-inducible genes that play significant functions in antiviral, cell proliferation, differentiation, apoptosis, and immune regulation functions. Previous studies have generally studied IFN-inducible genes in DM muscle tissue, but fewer have been conducted on PM tissues [13, 14, 17]. Recently, RNA sequence analysis of muscle biopsies in patients with DM and other forms of myositis (not including PM) suggested IFN1- and IFN2-inducible genes are differentially activated in various kinds of myositis, especially in DM [8]. Moreover, the role of IFN-inducible genes in PM and DM blood samples is controversial [15, 77, 78]. Therefore, bioinformatics analyses are needed to aggregate the findings of previous studies. This investigation showed that IFN-inducible genes are related to PM and DM by analyzing the data of previously performed transcriptome microarrays.

Another important finding of our study is the role of immune cell infiltration in PM/DM. While immune infiltration has been well studied in other autoimmune diseases, it had not yet been studied in PM/DM. We utilized CIBERSORT to perform a widespread assessment of the immune microenvironment in PM/DM, discovering elevated levels of macrophage infiltration, while resting Tregs infiltrated less; these findings may be correlated to the progression of PM/DM. Previous studies on macrophage differentiation patterns in PM/DM have demonstrated that DM and PM have different activation patterns and macrophage distribution. Specifically, in PM patients, macrophages activated in the early stage were mainly located in the myointima, which was the dominant histological manifestation. Whereas in DM, macrophages activated in late stages mainly appeared in the perifascicular area [79]. Other studies on macrophages and PM/DM have mainly focused on the activated receptors on the macrophage surface, such as CD206 and CD163. These studies have shown that activated macrophages are strongly correlated with the poor prognosis and mortality of PM/DM [80–82]. Another concern is that macrophage activation syndrome is caused by phagocytosis of hematopoietic components by activated macrophages. Macrophage activation syndrome is more common in RA and SLE; however, it has also been reported in some cases of DM [83, 84]. The above reports combined with our results have illustrated that macrophage infiltration is vital in the occurrence and progression of PM/DM.

The purpose of this investigation was to appraise biomarkers of PM/DM and to further investigate the function of immune cell infiltration in PM/DM. Our research has some limitations. First, we did not conduct *in vivo* tests to verify these outcomes. Second, we need to further study the definite mechanism of immune response caused by the five hub genes. Third, the CIBERSORT method is based upon finite genetic data, which might diverge from the heterogeneous interrelations of cells, characteristics of diseases, or plasticity of phenotypes. Finally, Mariampillai et al. [85] used unsupervised multiple correspondence analysis and hierarchical clustering analysis to aggregate patients by a database of the French myositis network according to myositis-specific antibodies, transcriptomic signatures, and clinicopathological correlations. They reclassified IIMs into four subgroups: DM, IMNM, ASS, and IBM [85, 86] and put forward the view that patients with PM were mainly present in IMNM and ASS clusters, and the use of PM should probably be discontinued. According to the latest classification, the PM patients we selected may be mixed with IMNM and ASS patients. However, as the original data of the five GEO series were not serologically grouped and discussed, it is not clear whether PM patients had a mixture of IMMN and ASS. Moreover, we did not explore the association of the five hub genes with the serological phenotypes (autoantibody profiles) of PM/DM patients. Although bioinformatics can reveal the internal mechanism, the results of our study need to be further validated by *in vivo* and *in vitro* tests and medical analysis.

We have comprehensively supplied a profound understanding of the overall molecular changes in the pathological mechanism of PM/DM and recognized five hub genes as potential therapeutic targets, including *TRIM22*, *IFI6*, *IFITM1*, *IFI35,* and *IRF9*. Moreover, through functional enrichment and pathway analysis, we discovered that these DEGs were generally enriched in immune function and the IFN signaling pathway. The WGCNA method identified MEblue and MEturquoise as key target modules in PM/DM. To our knowledge, this is the first study to focus on the role of immune cell infiltration in PM/DM. Additionally, we found that macrophage infiltration is influential in the occurrence and progression of PM/DM.

## Supplementary Material

Supplementary Figures

Supplementary Table 1
