# Supplementary material for: Identification of hub biomarkers and immune cell infiltration in polymyositis and dermatomyositis
Source: Aging (Albany NY). 2022 May 24;14(10):4530–55. doi: 10.18632/aging.204098 (PMC9186768; doi:10.18632/aging.204098)
Supplement: Supplementary Figures [file aging-14-204098-s001.pdf]

SUPPLEMENTARY FIGURES

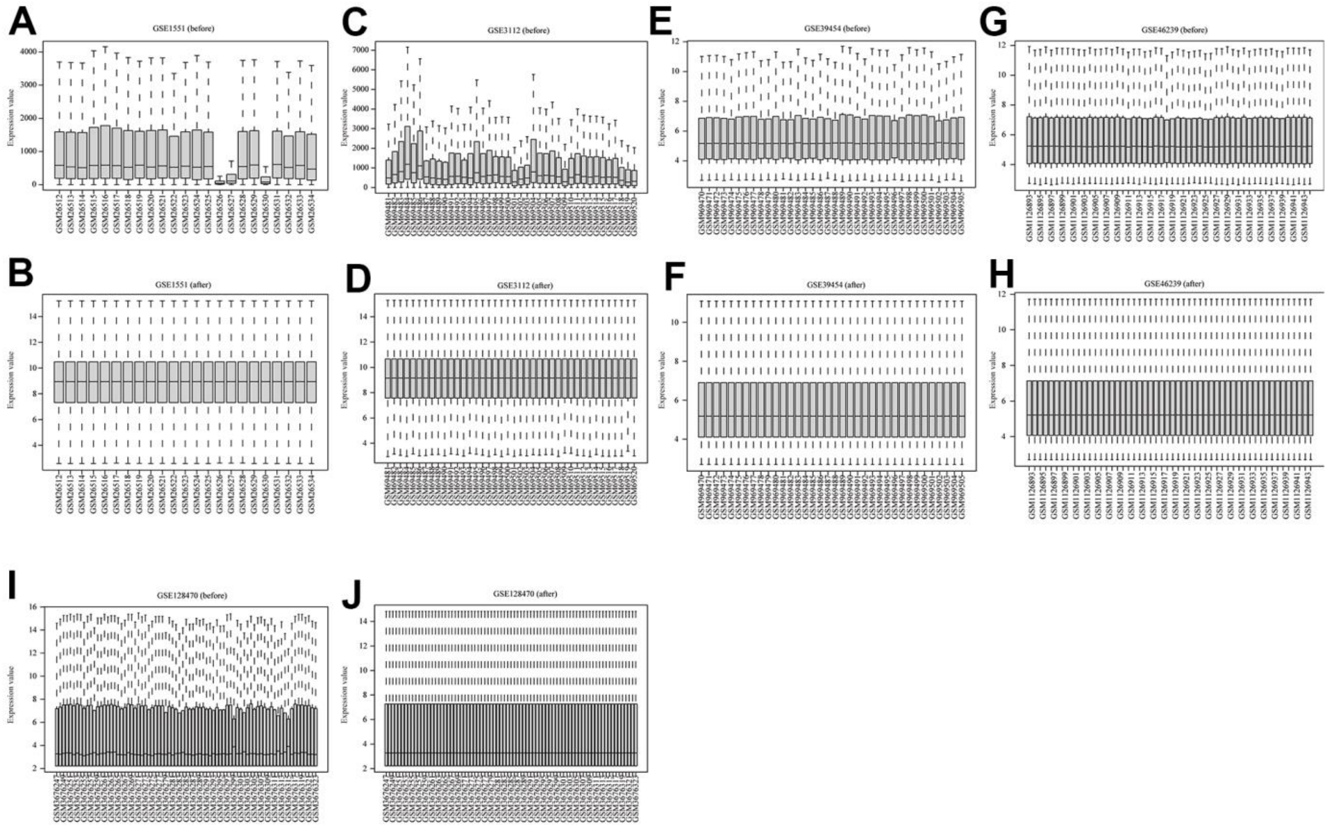

Supplementary Figure 1. Standardization of the five gene expressions. (**A**) Before normalization of GSE1551 microarray; (**B**) After normalization of GSE1551 microarray; (**C**) Before normalization of GSE3112 microarray; (**D**) After normalization of GSE3112 microarray; (**E**) Before normalization of GSE39454 microarray; (**F**) After normalization of GSE39454 microarray; (**G**) Before normalization of GSE46239 microarray; (**H**) After normalization of GSE46239 microarray; (**I**) Before normalization of GSE128470 microarray; (**J**) After normalization of GSE128470 microarray.

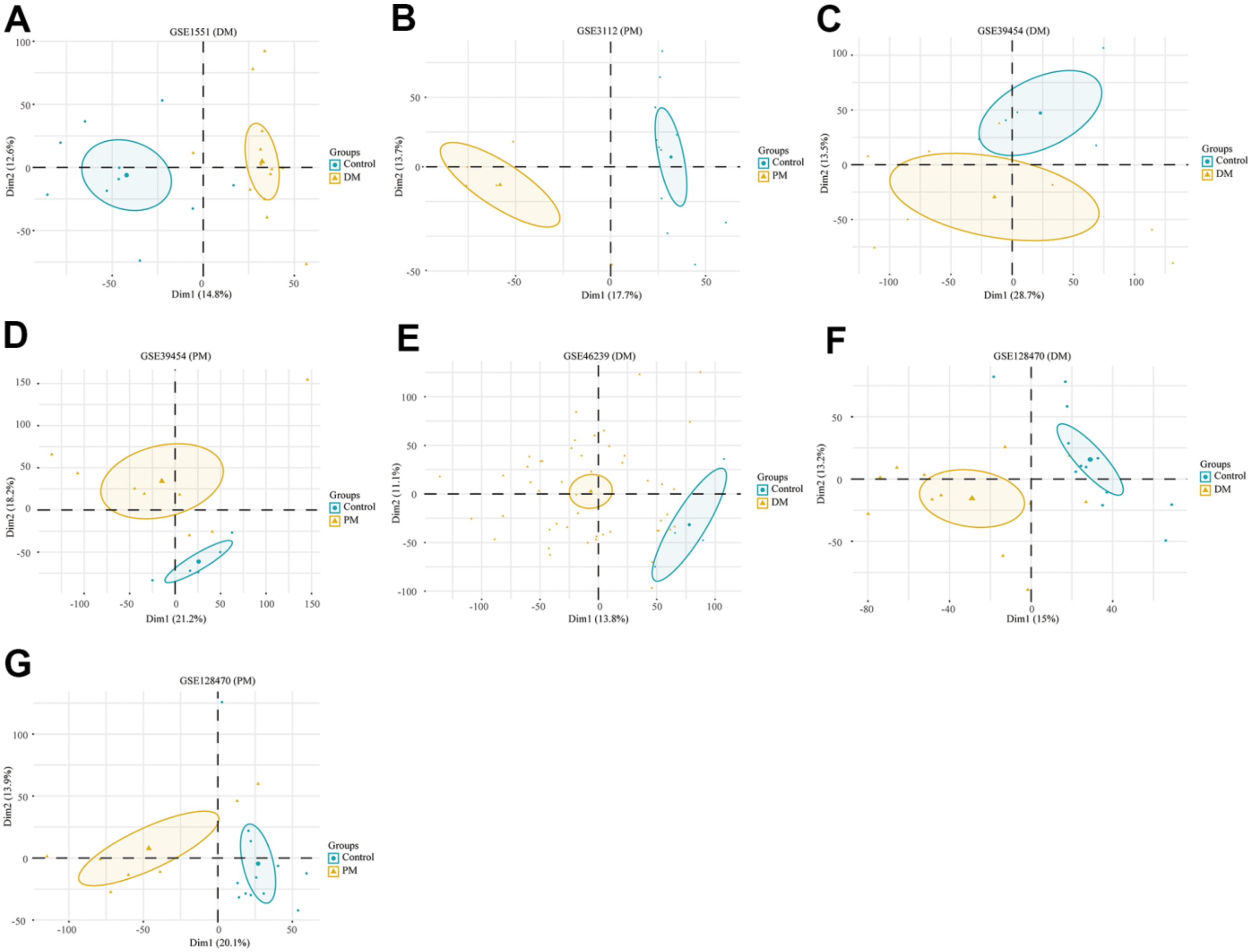

Supplementary Figure 2. Principal component analyses (PCA) performed on of seven data series. (**A**) GSE1551 (dermatomyositis, DM); (**B**) GSE3112 (polymyositis, PM); (**C**) GSE39454 (DM); (**D**) GSE39454 (PM); (**E**) GSE46239 (DM); (**F**) GSE128470 (DM); (**G**) GSE128470 (PM).

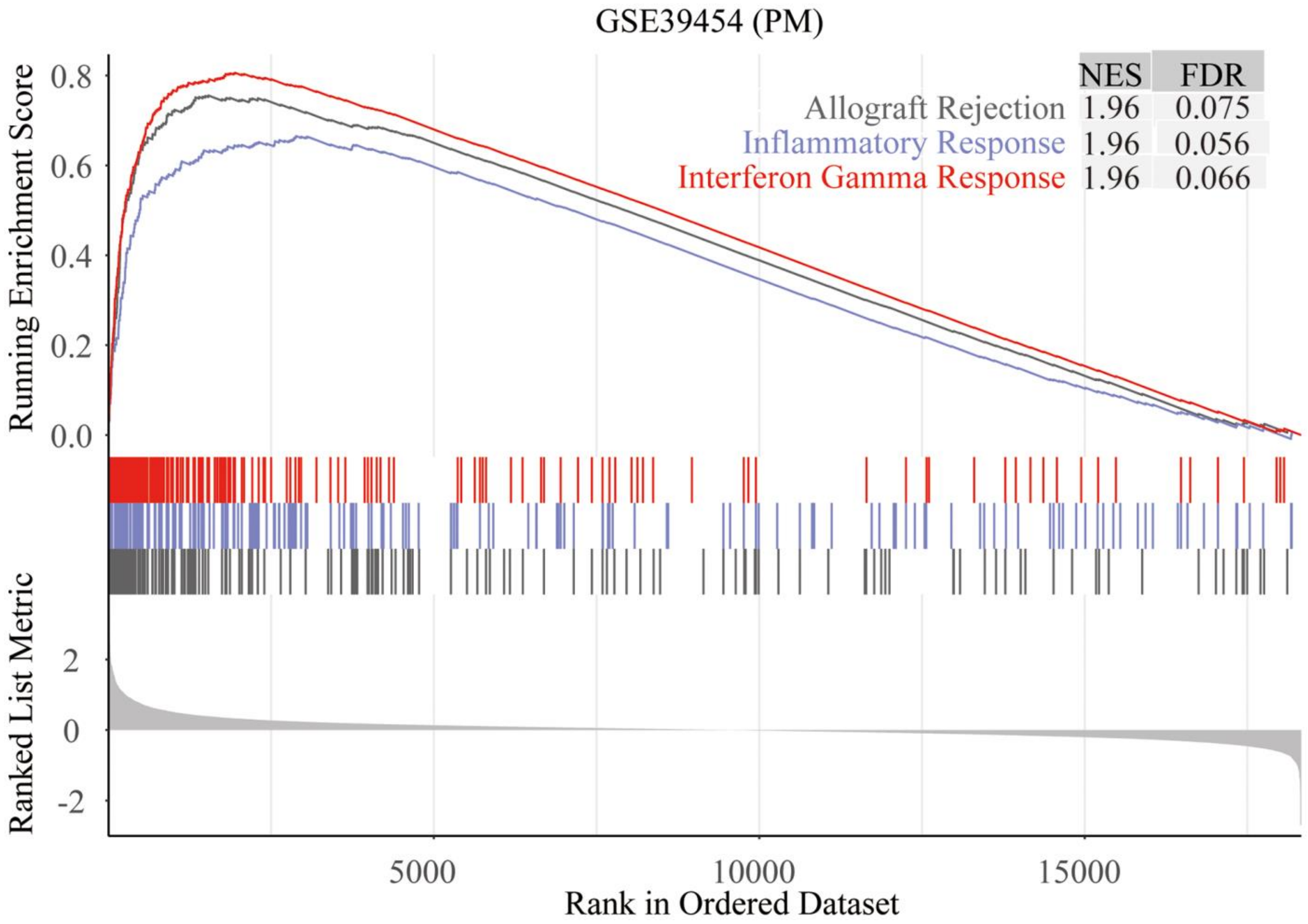

Supplementary Figure 3. Gene set enrichment analysis (GSEA) result of GSE39454 (PM).

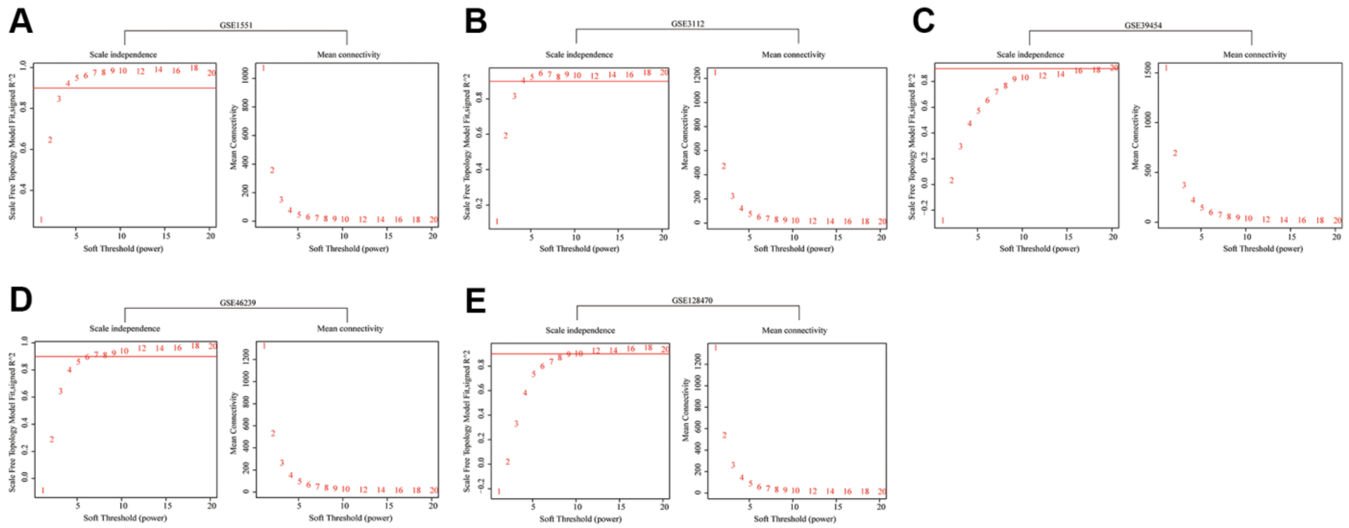

Supplementary Figure 4. Weighted gene co-expression network analysis (WGCNA) soft thresholds of the five microarrays. (**A**) GSE1551; (**B**) GSE3112; (**C**) GSE39454; (**D**) GSE46239; (**E**) GSE128470.

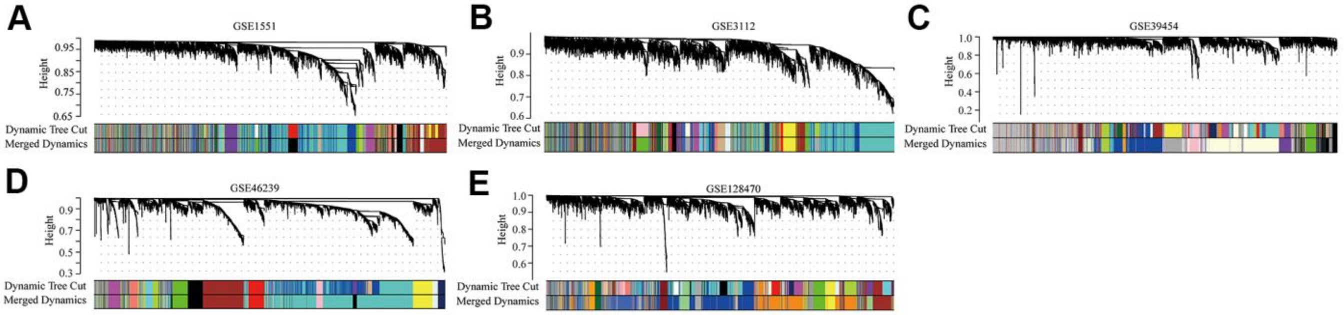

Supplementary Figure 5. WGCNA cluster dendrograms of the five microarrays. (**A**) GSE1551; (**B**) GSE3112; (**C**) GSE39454; (**D**) GSE46239; (**E**) GSE128470.

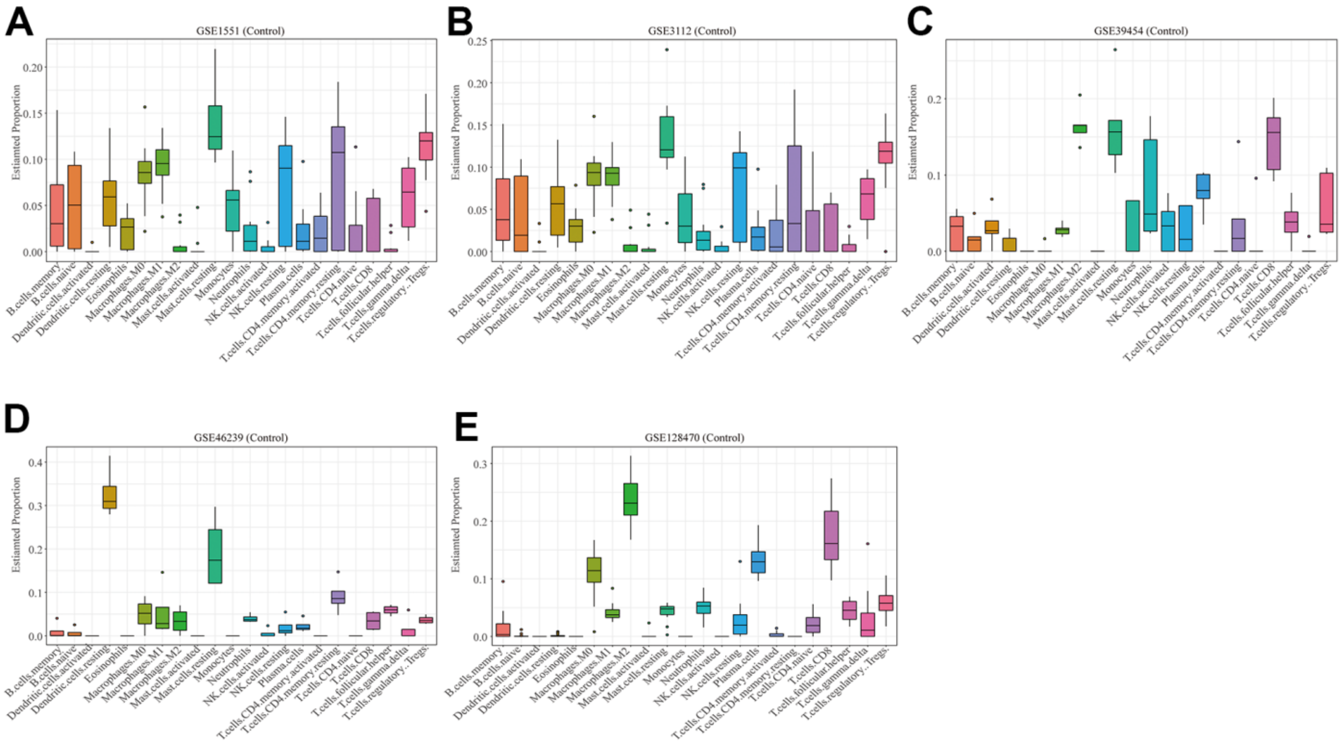

Supplementary Figure 6. Boxplots of the proportion of 22 immune cell sorts in healthy controls of the five microarrays. (**A**) GSE1551 (Control); (**B**) GSE3112 (Control); (**C**) GSE39454 (Control); (**D**) GSE46239 (Control); (**E**) GSE128470 (Control).

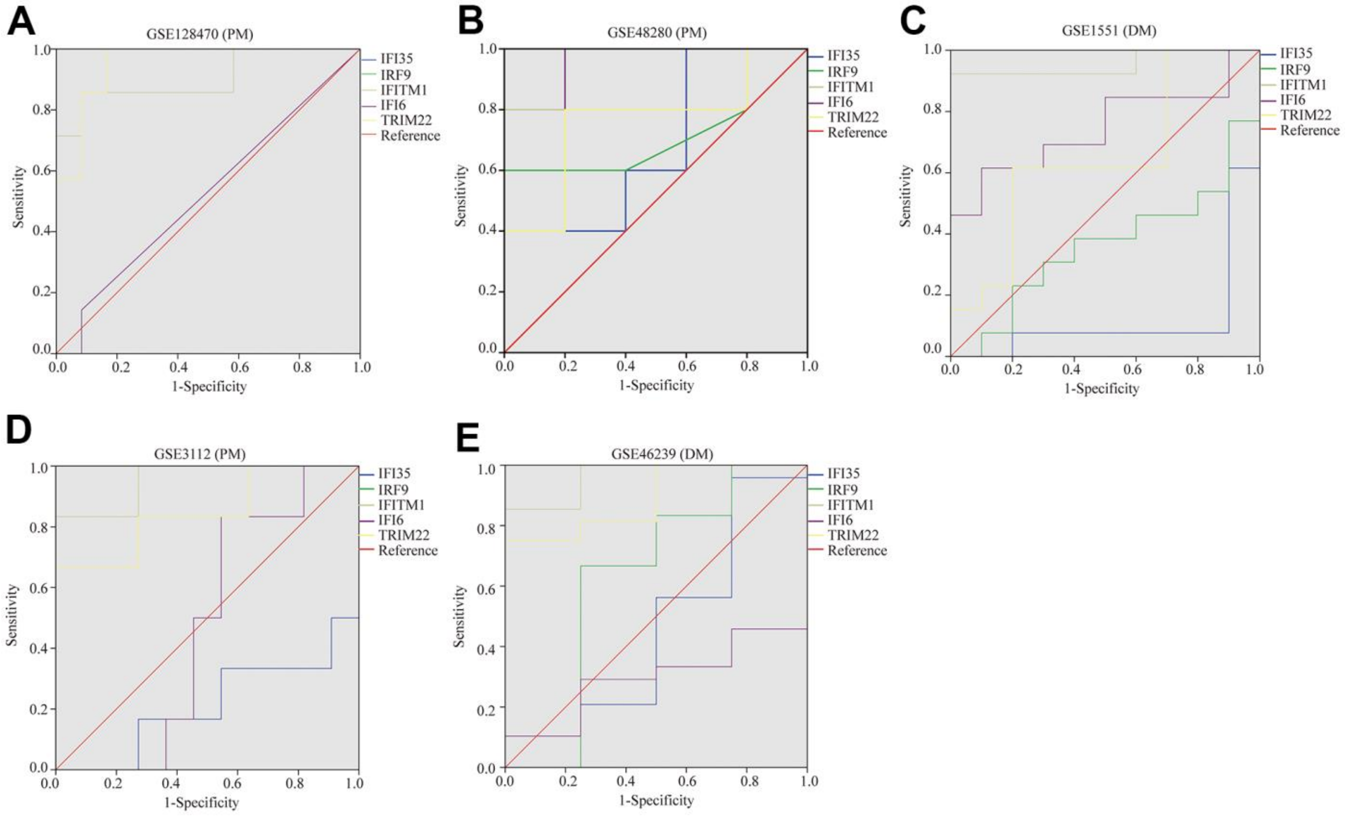

Supplementary Figure 7. Receiver operating characteristics of five hub genes. (**A**) GSE128470 (PM); (**B**) GSE48280 (PM); (**C**) GSE1551 (DM); (**D**) GSE3112 (PM); (**E**) GSE46239 (DM).
