# Supplementary material for: Identification of hub biomarkers and immune cell infiltration in polymyositis and dermatomyositis
Source: Aging (Albany NY). 2022 May 24;14(10):4530–55. doi: 10.18632/aging.204098 (PMC9186768; doi:10.18632/aging.204098)
Supplement: Supplementary Table 1 [file aging-14-204098-s002.pdf]

SUPPLEMENTARY TABLE

Supplementary Table 1. The 82 DEGs identified by RRA method.

Gene symbol	P value	Up/down regulated in DM/PM
ISG15	4.77E-08	up
C1QB	7.73E-08	up
HLA-A	9.33E-07	up
HLA-C	1.53E-06	up
HLA-B	2.15E-06	up
PSMB8	7.57E-06	up
C1QA	9.28E-06	up
GBP1	1.05E-05	up
UBE2L6	1.07E-05	up
OR7E47P	2.15E-05	down
PARP12	3.04E-05	up
IRF9	3.38E-05	up
LY96	5.85E-05	up
NMI	9.99E-05	up
IFIT3	1.69E-04	up
SLC15A3	1.76E-04	up
LGALS3BP	1.90E-04	up
IFI35	2.12E-04	up
IFITM3	2.27E-04	up
IFI27	2.28E-04	up
CHRNA1	2.63E-04	up
SIGLEC1	2.63E-04	up
TAP1	5.12E-04	up
TIMP1	6.93E-04	up
CXCL10	7.25E-04	up
IFITM1	8.10E-04	up
CDKN1A	8.63E-04	up
VAMP8	0.001155721	up
IFIT5	0.001253081	up
PSMB9	0.001357084	up
ZC3HAV1	0.001371369	up
CD163	0.001371369	up
HLA-J	0.00152631	up
PRUNE2	0.001846685	up
RARRES3	0.002123177	up
VCAM1	0.002561986	up
MYBPH	0.002561986	up
SP100	0.003358794	up
IFITM2	0.003358794	up
IFI6	0.003424865	up
AK055981	0.00382214	down
LY6E	0.004165117	up
IFI16	0.004177547	up
C1R	0.004677307	up
CCL2	0.005467811	up

GBP2	0.006409146	up
HLA-F	0.007232014	up
TMSB10	0.007643577	up
HLA-G	0.00845383	up
STAT1	0.008518256	up
MN1	0.008826432	down
VSIG4	0.009121644	up
CXCL9	0.00982952	up
IFI44L	0.010198991	up
PLSCR1	0.010662011	up
TYROBP	0.010970035	up
CCR1	0.011538863	up
PYCARD	0.011605535	up
C1S	0.014028206	up
PSME1	0.01403303	up
SAT1	0.015290515	up
MS4A4A	0.015378785	up
ATP2B2	0.016792652	down
SP110	0.017703687	up
NIPSNAP3B	0.018377843	down
OAS1	0.019487544	up
MX1	0.025685464	up
TRIM22	0.027970236	up
RHOBTB1	0.028239088	down
TM6SF1	0.028442829	down
OAS2	0.034005353	up
LAP3	0.034005353	up
MIF	0.034015527	up
CCL8	0.0361287	up
HERC5	0.040246026	up
PDP1	0.040568569	down
LMO1	0.040568569	down
KLF10	0.040568569	down
NREP	0.043705414	down
TFRC	0.044294258	down
RSAD2	0.045653159	up
RP4-781L3.1	0.045787271	up
